# Effectiveness of a worksite mindfulness-based multi-component intervention on lifestyle behaviors

**DOI:** 10.1186/1479-5868-11-9

**Published:** 2014-01-27

**Authors:** Jantien van Berkel, Cécile RL Boot, Karin I Proper, Paulien M Bongers, Allard J van der Beek

**Affiliations:** 1 Department of Public and Occupational Health - EMGO Institute for Health and Care Research, VU University Medical Center, van der Boechorststraat 7, 1081 BT, Amsterdam, the Netherlands; 2 Body@Work, Research Center on Physical Activity, Work and Health, TNO-VU University Medical Center, Amsterdam, the Netherlands; 3 Department of Work and Employment, TNO Quality of Life, Hoofddorp, the Netherlands

## Abstract

**Introduction:**

Overweight and obesity are associated with an increased risk of morbidity. Mindfulness training could be an effective strategy to optimize lifestyle behaviors related to body weight gain. The aim of this study was to evaluate the effectiveness of a worksite mindfulness-based multi-component intervention on vigorous physical activity in leisure time, sedentary behavior at work, fruit intake and determinants of these behaviors. The control group received information on existing lifestyle behavior- related facilities that were already available at the worksite.

**Methods:**

In a randomized controlled trial design (n = 257), 129 workers received a mindfulness training, followed by e-coaching, lunch walking routes and fruit. Outcome measures were assessed at baseline and after 6 and 12 months using questionnaires. Physical activity was also measured using accelerometers. Effects were analyzed using linear mixed effect models according to the intention-to-treat principle. Linear regression models (complete case analyses) were used as sensitivity analyses.

**Results:**

There were no significant differences in lifestyle behaviors and determinants of these behaviors between the intervention and control group after 6 or 12 months. The sensitivity analyses showed effect modification for gender in sedentary behavior at work at 6-month follow-up, although the main analyses did not.

**Conclusions:**

This study did not show an effect of a worksite mindfulness-based multi-component intervention on lifestyle behaviors and behavioral determinants after 6 and 12 months. The effectiveness of a worksite mindfulness-based multi-component intervention as a health promotion intervention for all workers could not be established.

## Introduction

Overweight and obesity are associated with an increased risk of morbidity and reduced life expectancy [1,2]. Further, they are associated with increased healthcare and medical costs [3,4]. In addition, overweight is associated with lower levels of productivity [5,6], higher rates of sick leave [7,8], and an increased risk of the need for a disability pension [9].

The development of overweight and obesity is the result of a complex interaction of social, economic, environmental and behavioral factors, on a background of genetic susceptibility. Considering the complexity, overweight and obesity is most often the result of gradual body weight gain, that is caused by an imbalance between energy expenditure (physical activity) and energy intake (dietary behavior) [10]. Besides a decrease of physical activity, sedentary behavior has been found to be independently associated with overweight and obesity [11]. As long-term consequences of overweight and obesity are burdensome for both individuals, employers and society, it is warranted to target lifestyle behaviors such as physical activity, dietary behavior and sedentary behavior in worksite health promotion interventions.

Studies evaluating the effect of worksite health promotion interventions targeting physical activity have shown that physical activity levels can be increased [12,13]. For example, moderate evidence was found for exercise training and active commuting interventions [13]. With respect to dietary behavior, a recent review showed limited to moderate evidence for a favourable effect of educational, environmental and multicomponent interventions [14]. To date, reviews of worksite interventions aimed at reducing sedentary behavior do not yet exist, probably because this field of research is relatively new. Controlled trials on feasibility and effectiveness of worksite interventions to reduce and break up sedentary behaviors at the worksite are thus needed [15].

It has been hypothesized that a (worksite) mindfulness training is an effective strategy to improve lifestyle behaviors and prevent overweight and obesity [16,17]. According to Chatzisarantis & Hagger [17], the working mechanism of mindfulness for lifestyle behaviors is to positively moderate the intention-behavior relationship [17]. In other words, being more mindful leads to behaving as intended. Positive effects of mindfulness based interventions outside the workplace have been reported for weight loss [18,19], short term increases in physical activity [20], and decreases of food cravings, external and emotional dietary behavior [21]. To date, no studies have evaluated the effectiveness of worksite mindfulness training on physical activity, dietary behavior and sedentary behavior.

In the “Mindful ‘Vitality In Practice’ (VIP)” study, we developed a worksite intervention aimed at improving physical activity, dietary behavior and sedentary behavior of workers at two Dutch research institutes [22]. The intervention was developed in a systematic way, based on the Intervention Mapping (IM) approach [23]. The study population played an important role in the development. Based on a needs assessment, the following behaviors were selected to target in the study population; vigorous physical activity in leisure time, sedentary behavior at work and fruit and vegetable intake [22]. The main element of the intervention was a mindfulness training. The aim of the present study was to evaluate the effectiveness of a worksite mindfulness-based multi-component intervention on the selected behaviors; vigorous physical activity in leisure time, sedentary behavior at work and fruit and vegetable intake, and behavioral determinants among workers of two Dutch research institutes.

## Methods

### Design and sample size calculation

The effectiveness of the Mindful VIP intervention was evaluated in a Randomized Controlled Trial. Participants who gave informed consent were measured at baseline (T0), as well as 6 months (T1), and 12 months (T2) of follow-up. The study design and procedures have been approved by the Medical Ethics Committee of the VU University Medical Center, Amsterdam, the Netherlands. The effects of the Mindful VIP intervention have been evaluated on two types of outcome measures: mental health related outcomes (i.e. work engagement) and lifestyle related outcomes. Since these types of outcomes had a different theoretical background and approach, the results have been reported in two different papers. The current paper describes the results on the lifestyle related outcomes. The results on the mental health outcomes are presented elsewhere (PLOS One in press), as are the details of the randomized controlled trial design [22].

The sample size was based on finding an effect on work engagement, which was chosen as the primary outcome of the RCT. An effect of a 10% increase in mean score was expected to be relevant and feasible. With a power of 90% and a two-sided alpha of 5%, both groups needed 89 participants. Accounting for a loss to follow-up of 25% over 12 months, each group needed 119 workers at baseline, thus an initial total of 238 participants for the two groups. The sample size calculation has been described more extensively elsewhere [22]. (Trial registration number: NTR2199.)

### Participants

All employees from two Dutch research institutes were invited to participate, between April 2010 and November 2010. The two research institutes added up to a total of 1820 employees (1570 and 250 respectively). Employees were considered eligible if they had signed informed consent, were not on sick leave for more than 4 weeks, and were not pregnant at the time of recruitment. In total, 257 participants were included at baseline. Mean age of the study population was 46 years. Furthermore, the study population consisted for 67% of women. About 60% of the study population had a healthy weight (BMI between 18.5 and 25).

### Randomization and blinding

After baseline measurements, participants were individually randomized to either the intervention or control group, using a computer-generated randomization sequence. Blinding of the participants and the trainers was not possible. After randomization, the research assistant notified each participant by e-mail about the group he or she was allocated to. Participants in both groups received a link to an intranet web page, with information about the health- and vitality-related offers of the participating organisations. In addition, the intranet web page of the intervention group contained information about the intervention and intervention materials.

### Intervention

The total duration of the intervention was six months. The Mindful VIP intervention comprised 8 weeks of in-company mindfulness training with homework exercises, followed by 8 sessions of e–coaching. The weekly mindfulness training sessions took 90 minutes and were held in a room at the worksite in a group setting of 4 to 17 participants. They participated in their own time (not during paid working hours), but the timetable was adapted to working hours as much as possible (before working hours, around lunch time and after working hours). The homework exercises comprised a variety of formal (“body scan” meditation, sitting meditation) and informal exercises (small exercises, such as breathing exercises when starting up the computer, and grocery shopping mindfully) and took approximately 30 minutes per day on 5 days per week. Materials for this training consisted of 2 cd’s with guided meditation exercises and a booklet with examples of workplace situations, background and (workplace) exercises. A short overview of the mindfulness intervention program is presented in Table 1. The mindfulness training was led by four certified trainers. These trainers were all members of the Society of Mindfulness-Based trainers in the Netherlands and Flanders, which means they have followed a mindfulness trainer education that is recognized by this Society. The e-coaching was integrated into the mindfulness training and was adapted to the mindfulness context as much as possible. Kindness and awareness were key-elements. During the penultimate session, the participants were asked to write a Personal Energy Plan (PEP), setting goals for themselves, answering the central question: “What do I need to do, to feel well at work?”, using the techniques and exercises from the training. (For example: ‘to sit and meditate five times a week’, or ‘to concentrate on my breath before speaking up in a meeting’). They had to e-mail the PEP to the trainer before the last session and that marked the start of the coaching by e-mail. The trainers provided 8 e-coaching sessions, existing of positive feedback (kindness) on the PEP and answers to questions. Additionally, free fruit and snack vegetables were provided during 6 months. Furthermore, lunch walking routes, and a buddy-system were offered as supportive tools. Fruit was provided at the location where the training was held. Lunch walking routes were provided by an intranet webpage. The buddy system was incorporated in the mindfulness training: the training was given in group setting and, in addition, participants were asked to form pairs to discuss homework exercises and to keep in contact between the sessions. More details of the intervention and its development are described elsewhere [22].

**Table 1:** An overview of the training program

**Week**	**Theme**	**Homework formal exercises**	**Homework informal exercises (time in minutes per week)**
1	Training mindful attention	- Bodyscan (5 x 20 min p/week)	- Walking with mindful attention (3 min)
			- Eating (3 bites) with mindful attention (3 min)
			- Stop, sit and do nothing for 1 minute (5 x 1 min p/week)
			- Read the booklet (background information, working situations)
2	Gaining by stopping and exploring boundaries	- Bodyscan and or sitting meditation (5 x 20 min p/week)	- Logbook for (small) pleasant happenings (5 x 5 min p/week)
			- Meditation exercise to start the working day (5 x 3 min)
			- Meditation exercise to finish the working day (5 x 3 min)
3	Switching from doing to being	- Bodyscan (5 x 20 min p/week)	- Logbook for (small) unpleasant happenings (5 x 5 min p/week)
		- Breathing exercises (5 x 3 min/week)	- Standing meditation in front of the window (1 3 min p/week)
			- Eat a raisin/apple/.. with attention
			- Walk the stairs with attention
4	Vigor and balance	- Office yoga (5 x 20 min)	- Walking with mindful attention (3 min)
		- Breathing exercises (5 x 3 min/week)	- Meditation exercise to finish the working day (3 min)
			- Yoga balance exercise (3 min)
			- Meditation (breathing) exercise with moments of inspiration or vigor (3 min)
5	Inspiration for working and living	- Sitting meditation (3 x 20 min p/week)	- Value orientation exercise (1 x 20 min
		- Breathing exercise (3 min, each stressful or joyous moment)	- Guided meditation exercise “the tree” ( values) (1 x 20 min)
6	Maintaining your center in interpersonal relationships	- Sitting meditation or body scan at choice (5 x 20 min p/week)	- Set your mobile phone alarm daily on a random moment and stop for one minute to notice how you are doing (1 min)
		- Room for breathing exercise, and notice your needs (3 min, each stressful or joyous moment)	- Compliment a colleague, notice what happens, internally and externally (1 min)
			- Train a different sense each day (hearing, seeing, etc.) (5 x 1 min)
7	Handling habits	- Walking meditation (3 × 30 min p/week)	- Write your Personal Energy Plan: what do you need to feel well at work? How can you attain that?
			- Mindful grocery shopping (using senses)
			- ‘Awareness of intake’ exercise (information, light, computer, phone, food, drinks) (1 day)
8	Caring for yourself	- Free choice of previous exercises	- Personal Energy Plan

### Measurements

#### Vigorous physical activity in leisure time

Vigorous physical activity in leisure time was measured both subjectively and objectively.

The Short Questionnaire to Assess Health Enhancing Physical Activity (SQUASH) [24] was used to measure duration, frequency and intensity of work transportation, household activities, leisure activities and work activities over the time period of one week. The SQUASH has shown to be sufficiently reliable and valid in a sample of Dutch adults [24]; the Spearman correlation coefficient for total reproducibility was 0.58 (95% confidence interval 0.36-0.74), and the Spearman correlation coefficient for total relative validity in comparison with the accelerometer was 0.45 (95% confidence interval 0.17-0.66). Items measuring time spent on vigorous physical activity in leisure time (i.e. sports) were selected and used to calculate the total duration of vigorous physical activity in minutes per week.

Vigorous physical activity was also assessed in a randomly selected subgroup (n = 100), using an accelerometer (Tri-axis Acti trainer activity monitor, Actigraph) over the time period of one week. Data were scored and interpreted using ActiLife 6.5.2, (ActiGraph, Pensacola) and participants was asked to wear an accelerometer on the hip during a period of 7 consecutive days, except while sleeping, washing and swimming. Participants wore the same accelerometer at every measurement (T0, T1, T2). A valid day was defined as at least ten hours of wearing time [25]. A valid week consisted of at least three valid days. Non-wearing periods were determined by a threshold of more than 30 minutes with 0 counts/minute. An epoch duration of 60 seconds was used [25,26]. The recommended cut-off point of more than 5725 counts per minute was used to calibrate the data to the intensity level ‘vigorous’ [27,28]. Total time spent in this category in minutes per week was calculated by summing all valid time periods in that category, divided by the number of valid wearing days (resulting in minutes per day) as the number of wearing days may vary across participants, and consequently multiplied by seven (resulting in minutes per week).

#### Sedentary behavior at work

To assess sedentary behavior at work, the questionnaire was based on an instrument used in a previous study to assess time spent sitting while at work [29]. Participants were asked to estimate time spent in hours per day, and in days per week on the following sedentary activities at work: 1) computer use, 2) reading, 3) meetings, 4) phone use, and 5) other activities.

#### Fruit intake

Fruit intake was assessed by the Short Fruit and Vegetable Questionnaire [30], which is assumed to be adequately valid (range Spearman’s r = 0.79- 0.80 for repeated measurements in the control group) [31]. The questionnaire consists of six questions for fruit consumption. Participants marked the number of servings of fruit (pieces or glasses of unsweetened juice, which added up equally) they consumed and the number of days per week, in an average week. The weekly total was divided by seven (days a week) to calculate the mean number of servings per day.

#### Determinants of lifestyle behaviors

Determinants are considered ‘those factors that have been found to be associated with behaviors’ [23] p195. Key determinants of the targeted lifestyle behaviors were identified in the development of the intervention in three focus group interviews among the study population, in combination with literature [22]. The determinants perceived behavioral control, intention and perceived barriers were hypothesized to explain the working mechanism of mindfulness on lifestyle behaviors [17].

Perceived behavioral control consists of two elements: self-efficacy and controllability (i.e. the beliefs of the ability to carry out a certain behavior and control over the behavior) [32]. Both these elements were measured as recommended by Ajzen [32] on a seven-point Likert scale. The items and scales for perceived behavioral control for vigorous physical activity and fruit intake, and their psychometric properties are presented in Table 2. Internal reliability was acceptable (range Cronbach’s α = 0.67-0.76) at all 3 measurements (T0-T1-T2) for perceived behavioral control of vigorous physical activity. For perceived behavioral control of fruit intake however, internal reliability was unacceptable (Cronbach’s α < 0.5) at all 3 measurements. Therefore, the corresponding items have been analyzed separately.

**Table 2:** Measurement of behavioral determinants: items and psychometric properties

**Determinant**	**Item (on a 7-point Likert scale)**	**Cronbach’s α**		
		**T0**	**T1**	**T2**
Perceived behavioral control	Exercising vigorously for at least 3 times 20 minutes this week is for me (completely possible – completely impossible)	.76	.67	.70
Physical activity	If I wanted to, I could exercise vigorously for at least 3 times 20 minutes this week (I completely agree- I completely disagree)			
	How much control do you experience on the amount of vigorous physical activity you have this week? (no control – complete control)*			
	The extent to which I exercise vigorously this week is mostly up to me. (completely agree- completely disagree)			
Perceived behavioral control	Eating at least 2 pieces of fruit per day this week is for me (completely possible – completely impossible)	.40**	.28**	.30**
Fruit intake	If I wanted to, I could eat at least 2 pieces of fruit per day this week (I completely agree- I completely disagree)			
	How much control do you experience on the amount of fruit you eat this week? (no control – complete control)*			
	The extent to which I eat fruit this week is mostly up to me. (completely agree- completely disagree)			
Intention	I intend to exercise vigorously for at least 3 times 20 minutes this week. (completely agree- completely disagree)	.94	.90	.86
Physical activity	I try to exercise vigorously for at least 3 times 20 minutes this week. (completely agree- completely disagree)			
Intention	I intend to eat daily at least 2 pieces of fruit this week. (completely agree- completely disagree)	.96	.94	.93
Fruit intake	I try to eat daily 2 pieces of fruit this week. (completely agree- completely disagree)			
Perceived barriers (lack of time)	I do not have enough time to engage in vigorous physical activity at least 3 times for 20 minutes this week, because of my work. (completely agree- completely disagree)*	.71	.82	.76
Physical activity	I do not have enough time to engage in vigorous physical activity at least 3 times for 20 minutes this week, because of my family life/ social life. (completely agree- completely disagree)*			

*Negative items have been recoded for analyse.

**Cronbach’s alpha is unacceptable, even when items were deleted. Therefore, items have been analysed separately.

Behavioral intention was measured by 2 items on a seven-point Likert scale for both vigorous physical activity and fruit intake [32]. Psychometric qualities were good to excellent at all 3 measurements (T0-T1-T2) for both vigorous physical activity (range Cronbach’s α = 0.86- 0.94) and fruit intake (range Cronbach’s α = 0.93- 0.96) (Table 2). Perceived barriers reflects the perception of obstacles to perform a specific (desirable) behavior [33]. A lack of time is a quite commonly perceived barrier, which impedes individuals to engage in physical activity [34]. Perceived barriers for physical activity were measured on a seven-point Likert scale by 2 items as presented in Table 2. Internal reliability was acceptable (range Cronbach’s α = 0.71- 0.82).

### Covariates

At baseline, data on potential effect modifiers and confounders were assessed, including age, gender, education (assessed in two categories of highest completed education: higher vocational education/university; or other), marital status (assessed in two categories: married/significant other; single/divorced/widow/widower), weight status (assessed in two categories based on Body Mass Index (BMI): healthy body weight BMI < 25 kg/m^2^; overweight BMI ≥ 25 kg/m^2^); and for women: pregnancy during the follow-up period.

BMI is calculated by dividing body weight in kilograms by the square of the body height in meters. Body height and body weight were measured by trained research assistants. Body height was measured to the nearest 0.1 cm without shoes. Body weight was measured to the nearest 0.5 kg in participants wearing indoor clothing and no shoes, after emptying their pockets. Measurements were repeated to increase reliability. Cohen’s kappa for the repeated measures of weight ranged from 0.76 to 0.87, which can be considered satisfactory.

### Statistical analyses

We performed linear mixed effect models for each outcome measure with the outcome measures as the dependent variable, group (intervention vs. control group) as independent variable and time of follow-up measurements (T1: follow up at 6 months and T2: follow up at 12 months) as fixed factor, while adjusting for the baseline levels of the outcome measure. Data were analyzed according to the intention-to-treat principle implying that all participants were analyzed according to the condition (i.e. intervention or control) using the linear mixed effect models (available case analysis), which uses all available information of all participants to estimate means and covariances, even when the number of measurements per participant through time available is different (which is the case for missing data on one of the follow up measurements). In addition, linear regression analyses with complete cases on T1 and on T2 separately were conducted as sensitivity analysis, to test the robustness of the results of the primary analyses. All statistical analyses were performed using SPSS (Version 20, Chicago, USA). All aforementioned covariates were tested for confounding; a change of 10% in the effect size was considered a relevant confounder. In addition, they were checked for potential effect modification, by adding interaction terms to the regression model. In case of a significant interaction, stratified analyses were performed. A p-level of < 0.05 was considered to indicate statistical significance. All statistical analyses were performed using SPSS (Version 20, Chicago, USA).

## Results

**Figure 1:**
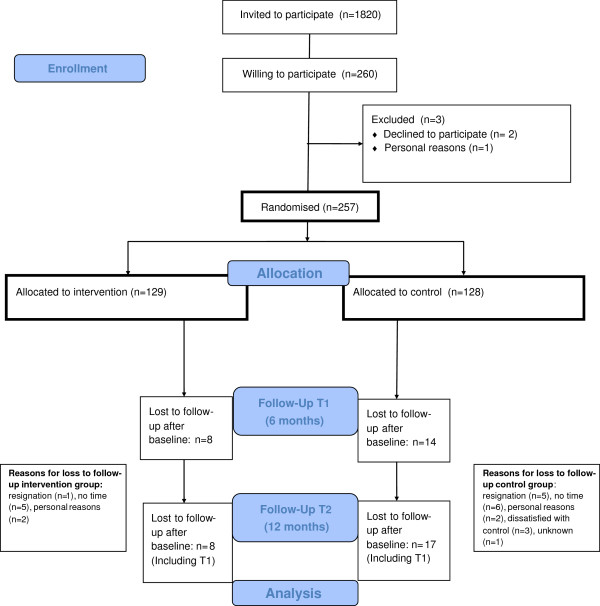
Flow chart.

As presented in the flow chart of this study (Figure 1), a total of 257 participants completed the baseline questionnaire and were randomized to the intervention (n = 129) or control group (n = 128). Between October 2010 and November 2011, the follow-up measurements took place. After 6 months, 231 participants completed the questionnaire and after 12 months 233 participants completed the questionnaire. Loss to follow-up after 12 months was 9.1% for the questionnaires and 16% for the accelerometers.

In Table 3, the characteristics of the study population are presented. It resulted from chi-square tests and t-tests that no significant differences existed between the intervention and control group characteristics at baseline. Both the intervention and the control group consisted mainly of highly educated workers (76.7% and 85.9%, respectively) and of women (63.6% and 71.1%, respectively). The majority of both groups had a healthy body weight (BMI < 25 kg/m^2^, 64.6% and 58.7%, respectively). On average, the intervention group worked 32.7 hours per week, and the control group worked 32.3 hours per week.

**Table 3:** Baseline characteristics of the study population (n = 257)

	**Intervention group (n = 129)**	**Control group (n = 128)**
Gender: Female, *%*	63.6	71.1
Marital status: Married or significant other, %	81.4	73.4
Education: Highly educated*, %	76.7	85.9
Age, year*s, M(sd)*	46.0 (9.4)	45.1 (9.6)
Contractual working hours per week, *M(sd)*	32.7 (5.4)	32.3 (5.5)
Overweight (Body Mass Index > 25),%	35.4	41.3

*Higher vocational education or university.

Table 4 shows means and standard deviations at baseline and follow-up measurements for the targeted lifestyle behaviors and their determinants. All average scores for behavioral determinants were quite low (overall range of averages: 1.3- 3.3), implying favourable scores. The means for perceived behavioral control for vigorous physical activity in leisure time ranged from 2.1 to 2.4 in both groups. Lowest means were scored for the separate items for self-efficacy and controllability of fruit intake (range 1.3-2.0) and highest means were scored for both intention of physical activity as intention of fruit intake (range 2.8-3.3).

**Table 4:** Means and standard deviations for lifestyle behaviors and behavioral determinants

**Lifestyle behaviors**	**Group**	**T0 M(sd)**	**T1 M(sd)**	**T2 M(sd)**
Vigorous physical activity (min/week) (Questionnaire)	I	69.2 (122.1)	56.4 (104.4)	39.4 (125.5)
	C	46.0 (123.1)	33.6 (70.4)	22.4 (80.1)
Vigorous physical activity (min/week) (Accelerometer subgroup)	I	13.6 (25.8)	8.9 (17.0)	10.4 (19.4)
	C	21.8 (45.5)	16.5 (26.4)	21.3 (30.3)
Sedentary behavior at work (min/week)	I	2311.3 (870.5)	2233.9 (693.0)	2249.9 (777.5)
	C	2295.7 (834.8)	2068.2 (611.0)	2212.8 (779.5)
Fruit intake (servings/day)	I	1.9 (1.0)	2.1 (1.0)	2.1 (1.1)
	C	1.9 (1.1)	1.9 (1.1)	1.9 (1.0)
Behavioral determinants*				
Perceived behavioral control for vigorous physical activity	I	2.1 (1.1)	2.4 (1.2)	2.2 (1.1)
	C	2.2 (1.3)	2.3 (1.2)	2.2 (1.2)
Eating at least 2 pieces of fruit per day this week is for me* (completely possible – completely impossible)	I	1.5 (1.0)	1.5 (1.2)	1.5 (1.1)
	C	2.0 (1.5)	1.9 (1.5)	1.6 (1.2)
If I wanted to, I could eat at least 2 pieces of fruit per day this week* (I completely agree- I completely disagree)	I	1.4 (1.2)	1.3 (0.8)	1.4 (1.1)
	C	1.4 (0.9)	1.4 (1.0)	1.4 (1.0)
How much control do you experience on the amount of fruit you eat this week?* (no control – complete control)**	I	1.6 (1.2)	1.7 (1.4)	1.6 (1.2)
	C	1.6 (1.3)	1.4 (0.9)	1.7 (1.3)
The extent to which I eat fruit this week is mostly up to me.* (I completely agree- I completely disagree)	I	1.8 (1.8)	2.1 (2.1)	1.7 (1.7)
	C	1.7 (1.6)	1.9 (2.0)	1.9 (1.8)
Intention for vigorous physical activity	I	2.8 (2.0)	3.2 (2.0)	2.8 (2.2)
	C	3.2 (2.3)	3.3 (2.3)	3.0 (2.3)
Intention for fruit intake	I	2.9 (2.0)	2.7 (2.0)	2.7 (2.0)
	C	3.1 (2.2)	3.1 (2.3)	3.1 (2.2)
Perceived barriers for vigorous physical activity**	I	2.4 (1.7)	2.5 (1.8)	2.4 (1.7)
	C	2.5 (1.8)	2.6 (1.8)	2.4 (1.7)

I= Intervention C=Control, *items were analyzed separately due to unacceptable cronbach’s alpha for the entire scale, **negative items have been recoded for analyses.

Table 5 shows the results of the primary analyses (linear mixed models) and sensitivity analyses (linear regression models) on lifestyle behaviors and their determinants. As the results of the crude and adjusted analyses did not differ, only the results of the crude analyses are presented. None of the potential confounders appeared to be of relevance in both primary and sensitivity analyses. The primary analyses (intention-to-treat analyses) did not show any effect modification. In the sensitivity analyses (complete case analyses), there was significant effect modification of gender for sedentary behavior at work at 6 months follow-up. From the stratified analyses on gender, it appeared that women in the control group sat 246 minutes per week less than women in the intervention group. There were no significant differences for men. No significant effects (p > 0.05) of the intervention were observed for the other lifestyle behaviors and behavioral determinants after 6 and 12 months.

**Table 5:** Intervention effects resulting from the primary analyses (linear mixed effect models) and sensitivity analyses (linear regression models) on lifestyle behaviors, and behavioral determinants after 6 (T1) and 12 months (T2), corrected for baseline (T0)

**Primary analyses**	**T1**			**T2**		
	**Fixed effects**	**P**	**95%CI**	**Fixed effects**	**P**	**95%CI**
Lifestyle behaviors						
Vigorous physical activity (min/week) (Questionnaire)	17.00	0.11	−3.96-37.96	−5.75	0.71	−35.73-24.23
Vigorous physical activity (min/week) (Accelerometer)	−1.07	0.69	−6.35-4.21	−2.93	0.46	−10.86-5.00
Sedentary behavior at work (min/week)	−12.87	0.86	−153.01-127.27	−113.45	0.26	−312.77-85.86
Fruit intake (servings/day)	0.03	0.70	−0.13-0.19	−0.02	0.86	−0.25-0.20
Behavioral determinants***						
Perceived behavioral control for vigorous physical activity	0.19	0.09	−0.03-0.42	−0.09	0.54	−0.41-0.22
Eating at least 2 pieces of fruit per day this week is for me (completely possible- completely impossible)*	0.01	0.91	−0.24-0.27	0.20	0.27	−0.16-0.56
If I wanted to, I could eat at least 2 pieces of fruit per day this week*	−0.09	0.40	−0.31-0.13	0.13	0.39	−0.17-0.45
How much control do you experience on the amount of fruit you eat this week?*,**	0.07	0.64	−0.21-0.35	−0.30	0.14	−0.71-0.10
The extent to which I eat fruit this week is mostly up to me.*	0.30	0.20	−0.16-0.76	−0.27	0.42	−0.92-0.39
Intention for vigorous physical activity	0.29	0.182	−0.13 – 0.71	−0.01	0.96	−0.62-0.59
Intention for fruit intake	−0.01	0.94	−0.36-0.34	0.07	0.79	−0.43-0.57
Perceived barriers (lack of time) Physical activity**	0.12	0.70	−0.21-0.45	0.06	0.82	−0.42-0.53
**Sensitivity analyses**	**T1**			**T2**		
	**b**	**P**	**95%CI**	**b**	**P**	**95% CI**
Lifestyle behaviors						
Vigorous physical activity (min/week) (Questionnaire)	14.74	0.16	−6.07-35.54	8.28	0.52	−16.90-33.47
Vigorous physical activity (min/week) (Accelerometer)	−6.21	0.19	−15.47- 3.06	−8.49	0.10	−18.52 – 1.54
Sedentary behavior at work (min/week)	146.87	0.07	−13.75-307.49	28.75	0.77	−164.33- 221.83
Fruit intake (servings/day)	0.14	0.13	−0.04-0.31	0.15	0.16	−0.06-0.37
Behavioral determinants***						
Perceived behavioral control for vigorous physical activity	0.18	0.21	−0.10-0.45	0.10	0.47	−0.16-0.35
Eating at least 2 pieces of fruit per day this week is for me (completely possible- completely impossible)*	−0.05	0.77	−0.36-0.26	0.07	0.63	−0.21-0.34
If I wanted to, I could eat at least 2 pieces of fruit per day this week*	−0.11	0.37	−0.34-0.13	0.03	0.84	−0.25-0.31
How much control do you experience on the amount of fruit you eat this week?*,**	0.30	0.06	−0.01-0.60	−0.01	0.95	−0.34-0.32
The extent to which I eat fruit this week is mostly up to me.*	0.18	0.51	−0.35-0.71	0.08	0.73	−0.55-0.38
Intention for vigorous physical activity	0.06	0.82	−0.44-0.55	0.08	0.75	−0.40-0.55
Intention for fruit intake	−0.36	0.13	−0.82-0.11	−0.30	0.18	−0.75-0.14
Perceived barriers (lack of time)Physical activity**	−0.04	0.86	−0.48-0.40	0.02	0.95	−0.39-0.42

CI= Confidence Interval *items were analyzed separately due to unacceptable cronbach’s alpha for the entire scale , **negative items have been recoded for analyses, ***on 7-point Likertscale, with lower scores representing more favorable determinant.

## Discussion

This study did not show an effect of a worksite mindfulness-based multi-component intervention on vigorous physical activity, fruit intake and behavioral determinants after 6 and 12 months. A significant interaction effect was found for gender and sedentary behavior at work, which had decreased significantly in women in the control group after 6 months. After 12 months there were no differences in sedentary behavior at work between women in intervention and control group.

A possible explanation for not finding the expected effects could be that we aimed at stimulating healthy behavior in a population without specific risks. Previous research on the effectiveness of mindfulness training on lifestyle behavior had study populations that were obese or overweight [18,19,21]. It might be that the mindfulness training as under study is less effective in populations not at risk, i.e. with a healthy body weight. In other words, it might be that ceiling effects occurred. This does however not imply that mindfulness is not effective among this group, but that another type, intensity and duration of a mindfulness intervention might be effective. Due to lack of studies to the effect of mindfulness among relatively healthy persons, future research is relevant from a population health approach. Serious health consequences (such as all-cause mortality) associated with body weight, usually start with a BMI higher than 30 [35]. Our study population consisted of very few participants with a BMI higher than 30, which made itimpossible to perform a subgroup analysis to explore the effectiveness in a high risk subgroup.

Another possibility of ceiling effects might have occurred in the behavioral determinants.Although the targeted lifestyle behaviors were selected on their room for improvement (for example, 60.1% of the study population did not engage in vigorous physical activity at baseline at all; and the recommended target of 2 pieces of fruit per day was not the average consumption at baseline [15]), it appeared that the scores on the key determinants were already close to optimal. This implies that there was no room for improvement (so-called ceiling-effect), even though the corresponding behaviors seem susceptible to change. It might be that the measurement of the determinants was not sensitive enough to distinguish small changes, which was especially important since the population under study was relatively healthy.

Next to the aforementioned explanations, the timing of the measurements could also be relevant to explain the lack of effect. Other studies evaluating the effectiveness of mindfulness based interventions on lifestyle behaviors and weight-related outcomes found effects at the immediate follow-up measurement, that is immediately after the training [18,19,21]. Both Tapper and colleagues and Van Dalen and colleagues found reduced effects at the follow-up measurement at 6 months and 3 months respectively [18,19]. Kearny and colleagues [36], however, did not find any effects at their follow-up measurement of 4 months. It might be that the effects of the mindfulness training component in our intervention wore of before our first follow-up measurement took place, after the total intervention duration of 6 months.

To enhance fruit intake, participants in our intervention were offered free fruit at the workplace. This fruit was offered during the whole intervention period of six months. In contrast to our findings, other interventions showed significant effects on fruit intake following offering free fruit during 6 months [37]. Despite this, the fruit was well appreciated by the participants and the reach among participants was reasonably good (69%) [38]. Possibly, the participants who made use of the fruit at the workplace, were the ones who already ate fruit before the intervention and now ate the provided fruit instead of bringing their own.

The difference in effect modification between the primary analyses and the sensitivity analyses for sedentary behavior at work after 6 months is probably caused by the difference in handling missing data. The primary analyses concerned intention-to-treat analyses, whereas the sensitivity analysis concerned complete-case-analyses. For the sedentary behavior at work questions, there were relatively more missing values than for the other questions (T1: 18% missing and T2: 19% missing). Therefore, we believe that the effect modification for gender we found in the sensitivity analyses was a Type 1 error, especially given the number of analyses performed. In addition, the use of a questionnaire that had not been validated, though more often used in worksite health promotion intervention studies, may have contributed to less reliable findings. Therefore, the results should be interpreted with caution.

### Strengths and limitations

The major strength of this study is that it is the first study to examine the effectiveness of a worksite mindfulness-based intervention targeting lifestyle behaviors in a randomized controlled trial design, which is the most reliable design for intervention studies. Second, the number of participants is quite large compared to other studies on the efficacy of mindfulness interventions on lifestyle behaviors, overweight and obesity (range n = 12–84) [18,19,21,36,39]. In addition, the duration of follow-up was 12 months. Long term effectiveness is especially important for interventions aimed at behavioral change, since sustaining a changed behavioral pattern is difficult, especially for weight maintenance [40]. Another strength, is that the intervention was tailored for the target population. In addition, loss to follow-up was very limited (less than 10%). A last strength, is that we measured physical activity objectively using accelerometers in a subgroup in addition to subjectively using a questionnaire.

A first limitation of this study, is the lack of an immediate post-intervention measurement of the program component mindfulness training. Long term effects are relevant, but when an immediate effect is present, this implies that the maintenance of the direct effects deserve attention, rather than focussing on changing the intervention component itself.

Another limitation of this study - given the complexity of overweight and obesity- is that environmental factors at the participating research institutes have not been taken into account. A meta-analytic review showed that worksite physical activity and dietary interventions containing an environmental component were more effective than individual interventions [41]. Examples of an environmental component are a construction design where the stairs are more easily to find then elevators, sit-stand work stations, or standing meeting facilities.

### Implications for research and practice

For the future development of worksite health promotion interventions, it is recommended to assess the selected key determinants on the potential for improvement among the study population, next to the potential effects of selected behaviors. Furthermore, it is recommended for multi-component intervention studies to perform intermediate measurement, that is, after a single component, to gain insight in the possible attrition of effects, if present.

This study aiming at health promotion for all workers at two research institutes did not show any effects. For future mindfulness research, intervention aims besides the cognitive dimension of lifestyle behavior could be explored. Given the complexity of overweight and obesity, it is recommended for worksite health promotion to also address other dimensions and combine environmental and individual components in an intervention. In addition, it is recommended to develop and validate a reliable questionnaire to measure sedentary behavior at work, which to date does not yet exist.

## Conclusion

This study did not show effects of a worksite mindfulness based intervention on vigorous physical activity, fruit intake and behavioral determinants after 6 and 12 months among a group of relatively highly educated workers. Thereby, the results do not support the implementation of the mindfulness intervention as evaluated in this study among this group.

## Competing interests

The authors declare that they have no competing interests.

## Authors’ contributions

All authors contributed to the conceptual design of the study and intellectual input into the design of this paper. JvB performed data collection, analysed data and drafted the manuscript. All authors contributed to the writing of the manuscript. All authors read and approved the final manuscript.
